# Discrimination between the Triglyceride Form and the Ethyl Ester Form of Fish Oil Using Chromatography–Mass Spectrometry

**DOI:** 10.3390/foods13071128

**Published:** 2024-04-08

**Authors:** Mingxuan Liu, Yuchong Liu, Xiupin Wang

**Affiliations:** 1 Oil Crops Research Institute, Chinese Academy of Agricultural Sciences, Wuhan 430062, China; liumx159@163.com (M.L.); 15972690571@163.com (Y.L.); 2 College of Chemistry, Beijing University of Chemical Technology, Beijing 100029, China; 3 Key Laboratory of Biology and Genetic Improvement of Oil Crops, Ministry of Agriculture, Wuhan 430062, China; 4 Key Laboratory of Detection for Mycotoxins, Ministry of Agriculture, Wuhan 430062, China; 5 Laboratory of Risk Assessment for Oilseeds Products (Wuhan), Ministry of Agriculture, Wuhan 430062, China; 6 Quality Inspection and Test Center for Oilseeds Products, Ministry of Agriculture, Wuhan 430062, China

**Keywords:** chromatography, mass spectrometry (MS), discrimination, triglyceride, ethyl ester, fish oil

## Abstract

Although the triglyceride form is the natural form of fish oil found in fish, the ethyl ester form of fish oil, which is used during processing to save costs, is also present on the market. In this study, fatty acids and lipids were determined using gas chromatography–mass spectrometry (GC–MS) and liquid chromatography–linear ion trap mass spectrometry (LC–LIT/MS), respectively, according to developed methods. The identification of fatty acids was based on the mass spectral characteristics and equivalent chain lengths. However, the fatty acid contents of both forms of fish oils are quite similar. The application of the LC–LIT/MS method for the structural characterization of triacylglycerols (TAGs) and the mechanism of LIT/MS fragmentation are also discussed. Neutral losses of CH_2_=CH_2_ (*m*/*z* 28) and CH_3_CH_2_OH (*m*/*z* 46), which are LIT/MS characteristics of ethyl ester from fish oil, were found for the first time. The triglyceride form of fish oils was easily and accurately identified using fingerprint chromatography. In conclusion, lipid analysis combined with LC–LIT/MS showed an improved capability to distinguish between types of fish oil.

## 1. Introduction

Fish oil is one of the most popular functional supplements, providing human beings with essential nutritional components and the omega-3 polyunsaturated fatty acids eicosapentaenoic acid (EPA) and docosahexaenoic acid (DHA), which cannot be synthesized by the human body [1,2]. A large study showed that EPA and DHA might benefit those with cardiovascular disease [3,4] and cancer [5,6]; improve memory [7,8], eyesight [9,10], and immune function [11,12]; and decrease the effects of the systemic inflammatory response [13,14]. EPA and DHA dietary reference intakes were first recommended by the United Nations Food and Agriculture Organization (FAO) and the World Health Organization (WHO) in 2008. Relevant statistical data show that China’s current mean EPA and DHA intake (≈37.6 mg/day) is much lower than currently recommended (250–2000 mg/day) [15,16]. Therefore, increasing the intake of fish or fish oil is the best way to supplement DHA and EPA. However, the Chinese market is inundated with fake and poor-quality commodities that compete with the most popular and best-selling fish oils, which not only results in consumer fraud but also endangers the lives and health of humans. There is a great demand for an effective method of quality assurance. In this study, we selected a triglyceride form of fish oil (TGFFO) and an ethyl ester form of fish oil (EEFFO) as target forms and analyzed their possible nutritive composition to establish a reliable method for distinguishing between the two forms of fish oil.

The fat in fish oil products is mainly in the form of ethyl ester, triglycerides (TG), free fatty acids (FFAs), and phospholipids (PLs), but EEFFO and TGFFO are the most common products in domestic markets [17,18]. Because its molecular structure is very close to natural fish oil, TGFFO is easier for humans to digest and absorb than EEFFO. Meanwhile, the fatty acid ethyl ester, due to its decomposition into ethanol in the gastrointestinal tract, will lead to undesirable consequences, especially in older adults and children [19,20]. The incubation of cells with 600 µmol/L ethyl oleate or 800 µmol/L ethyl arachidonate decreased [methyl-3H]thymidine incorporation into HepG2 cells by 31% and 37%, respectively [21]. Thus, the price of TGFFO is much higher than that of EEFFO in the international market. It is imperative to thoroughly analyze the composition of fish oil for a comparative analysis of the two different forms of fish oils.

Different analytical methods are used to analyze the inorganic and organic constituents of fish oils. However, lipids are the main components of fish oils. Fatty acids (FA), as part of the lipid fraction, are a principal component of fish oils. This study focuses on the analysis of lipids and fatty acids using multiple combined methods to distinguish between EEFFO and TGFFO.

Fatty acids, as functional monomers in fish oil, are typically analyzed using gas chromatography techniques [22,23,24,25]. To make the results more accurate, the steps related to the derivatization of fatty acids are usually required before separating them by gas chromatography. Thurnhofer et al. developed a methodological system for the analysis of fatty acid methyl esters (FAMEs) using gas chromatography and analyzed the fatty acid methyl esters (FAMEs) from sunflower oil, plate oil, and cod liver oil, respectively. The extent of rationalization of the application of the method was confirmed [26,27]. In a previous study, it was demonstrated that gas chromatography-flame ionization detection (FID) analysis was suitable for the FA profiling of agricultural products such as different mustard varieties [28]. Compared to GC–FID, GC–MS could provide more structural information. Ferracane et al. used a rapid gas chromatography–mass spectrometry method to analyze fatty acid methyl esters in blood samples and dietary supplements, and improved the detection and analysis system of the above fatty acid methyl ester derivatives [29]. While Zhang et al. made a new breakthrough in the separation and extraction of fatty acids from channel catfish muscle samples, the samples were extracted by supercritical carbon dioxide fluid extraction using gas chromatography–electron ionization–mass spectrometry, which demonstrated good sensitivity and reproducibility [30]. In addition, Retrato et al. used a direct GC–MS method to identify 22 fatty acids with varying chain lengths and degrees of unsaturation, respectively, which is widely used in the field of diverse assessment of total parenteral nutrition (TPN) product quality [31]. Sun et al. used gas chromatography–mass spectrometry to analyze the fatty acid profiles of flaxseed oil in selected ion monitoring modes, and analyzed the data and built models through multivariate analysis such as principal component analysis (PCA) and the recursive support vector machine (R-SVM) [32]. Moreover, GC–MS has well-established databases for FA identification with higher efficiency and better selectivity compared to GC–FID. As a result, GC–MS is the most frequently used method for fatty acid analysis. In our paper, fatty acids were identified using the equivalent chain length (ECL) values and MS characteristics and were quantified by the peak area normalization method. Further, new liquid chromatography/mass spectrometry [33] and comprehensive gas chromatography [34] analytical methods have also been used for the detection of fatty acids, but these approaches are less frequently applied.

Triglycerides and ethyl esters are the main components of TGFFOs and EEFFOs, respectively. The most common method of triacylglycerol (TAG) detection is high-performance liquid chromatography–mass spectrometry (HPLC–MS) in oils [35,36,37]. Indelicato et al. characterized triglycerides (TAGs) qualitatively and quantitatively in crude tuna fish oil using HPLC–MS [38]. Finally, 81 TAGs were identified preliminarily, but the quantitative results are not complete. Song et al. completed the quantitative analysis of eicosapentaenoic acid/docosahexaenoic acid (EPA/DHA) lipid composition of fish oils using ultra-performance liquid chromatography–electrospray ionization tandem mass spectrometry (UPLC-ESI-MS/MS), but less for the structural qualitative aspects [39]. Krill oil is another representative source, and Castro-Gómez et al. identified triglycerides in krill oil using multiple detection techniques, including gas chromatography–mass spectrometry/flame ionization detector (GC–MS/FID), a fast spectrometry–evaporative light scattering detector (FC-ELSD), and HPLC–ELSD, respectively. Due to the large number of lipid species identified, no study was initiated to quantify the lipid fractions of krill oil [40]. Although the analysis of the fatty acid ethyl ester has not been as much of a concern as fatty acids and TAGs in previous studies, several analytical technologies can be used for detection. For example, Fourier transform near-infrared spectroscopy (FT–NIR) principles and instrumentation [41], solid-phase microextraction and GC–MS [42,43], and HPLC–MS [44,45] have been used to analyze ethyl ester qualitatively.

In this study, fatty acids in two different forms of fish oils were analyzed using GC–MS, and 22 fatty acids were identified and quantified, with similar fatty acid contents in all seven brands of fish oil samples. TAGs and the fatty acid ethyl ester were separated using reverse-phase liquid chromatography and detected using atmospheric pressure chemical ionization (APCI) MS in positive ionization mode to compare the fingerprint chromatography of the two different fish oils, and a considerable difference was found between the two. As a result, the characteristics of TGFFO and EEFFO were identified using LC–MS, and we were able to effectively discriminate between the two fish oils.

## 2. Materials and Methods

### 2.1. Materials and Reagents

Seven brands of fish oil samples were purchased from official pharmacies to ensure the quality of the selected fish oils (Table 1). Before the experiment, all samples were serially numbered and stored in a refrigerator (below 4 °C). Solvents were purchased from J&K Chemical, Ltd. (Shanghai, China). The anhydrous diethyl ether, potassium hydroxide, ethanol, and petroleum ether (boiling range: 60–90 °C) were of analytical grade, and the N-hexane, isopropyl alcohol, methanol, and acetonitrile were of HPLC grade. Ultrapure water was prepared using a Milli-Q System (Millipore, Billerica, MA, USA). A 37-component FAME mix was purchased from Sigma (St. Louis, MO, USA). Ethyl oleate (18:1 EE, purity ≥ 98%) was purchased from Sigma Aldrich (St. Louis, MO, USA). KOH–CH_3_OH (0.4 mol/L) was prepared in our laboratory by dissolving reagent-grade KOH in methanol.

### 2.2. Methods

#### 2.2.1. Fatty Acid Analysis

The fatty acid analysis process includes sample preparation, GC–MS analysis, identification, and quantification of fatty acids.

##### Sample Preparation

Fatty acid analysis typically involves weighing samples, derivatization, and GC–MS analysis. The fatty acids in the fish oil samples were first methylated to fatty acid methyl esters (FAME) and then analyzed by GC–MS. Fish oil samples (60 mg) were transferred into 10-mL screw-capped polypropylene centrifuge tubes, and 2 mL of an anhydrous diethyl ether:petroleum ether mixture (1:1, *v*/*v*) and 1 mL of a KOH–CH_3_OH mixture (0.4 mol/L) were added. The tubes were vortexed for 30 s and allowed to rest for 2.5 h. Then, 2 mL of ultrapure water was added, vortexed for 30 s, and centrifuged at 4500 rpm for 2 min. Next, 200 μL of the organic layer and 800 μL of petroleum ether were transferred to a 2-mL sample vial and subjected to GC–MS analysis [32].

##### GC–MS Analysis

The GC–MS system included an Agilent GC–7890 gas chromatograph interfacing with an Agilent 5973 mass spectrometer. Chromatographic separation was performed on a DB-23 fused silica capillary column (30 m × 0.25 mm i.d. 0.15-μm film, Agilent Technologies). The Agilent GC–7890 had the following configuration. Helium (99.999% purity) was used as the carrier gas at a flow rate of 1.2 mL/min. The temperature range of the column was increased from 100 °C (held for 0.2 min) to 215 °C at a rate of 10 °C/min and held for 0.1 min. Then, it was increased to the final temperature of 224 °C at a rate of 2 °C/min and held for 0.2 min. The temperatures of the injector, ion source, and detector were 220, 250, and 150 °C, respectively. The mass spectrometric conditions were as follows: EI ionization mode, electron energy of 70 eV, and a solvent cut time of 3 min. The splitting ratio was 20:1. The selected ion monitoring (SIM) mode was *m*/*z* 55, 67, 74, and 79.

##### Identification of Fatty Acids

To identify fatty acids in fish oils, retention times and mass spectral characteristics were combined. The extracted ion chromatograms of *m*/*z* 74, 87, 55, 67, and 79 were used to identify all expected saturated FAMEs in the GC–MS data after background subtraction and denoising. Heuristic evolving latent projections (HELP) are reported as a method to resolve two-way bilinear multicomponent data into spectra and chromatograms of the pure constituents. To determine the pure chromatographic profile and pure mass spectrum of FAMEs, background-subtracted GC–MS data were resolved using the heuristic evolving latent project (HELP). In our method, we set the threshold signal-to-noise ratio of the peak to 3. An automatic mass spectrum search identifies all straight saturated FAMEs.

We then transferred the retention times of the expected and verified saturated FAMEs into ECL. As calibration series, straight saturated FAMEs were used, and using Equation (1), the ECL value of each fatty acid was calculated from the straight saturated FAMEs that eluted immediately before and after the compound of interest. Using straight saturated FAMEs as a reference, the ECL for unsaturated FAMEs can be calculated.

The fatty acids were identified using *ECL* values [33]. The 37-component FAME mix was used as an external standard to calibrate *ECL* data. The *ECL* value of each fatty acid was calculated using Equation (1).

(1) $$ECL(x)=n+\frac{RT(x)-RT(n)}{RT(n+1)-RT(n)}$$

Here, *n* and *n* + 1 represent the number of carbons on the straight saturated FAME eluting immediately before and after the compound of interest, respectively.

##### Quantification of Fatty Acids

The fatty acids were quantified by the peak area normalization method. Chromatography is based on quantitative analysis. The extracted ion chromatograms of *m*/*z* 74, 87, 55, 67, and 79 were used to quantify all expected saturated FAMEs based on the area of chromatographic peaks. Since the amount of a FAME is proportional to its peak area, the correction factor of each FAME in fish oils is similar, and the peak area normalization can be directly used for calculation. The corresponding formulas are presented in Equation (2).

(2) $$C\%=\frac{A_{i}}{A_{1}+A_{2}+\cdots +A_{n}}\times 100\%$$

Here, *C* represents the content and *A* represents the peak area of each FAME.

#### 2.2.2. Analysis of TAGs and EEs

The analysis of TAGs and EEs process includes sample preparation, LC-MS analysis, identification, and quantification of TAGs and EEs.

##### Sample Preparation

Fish oil samples from seven brands were examined, and 0.1 g of each fish oil sample was transferred into 10-mL screw-capped polypropylene centrifuge tubes. Then, 5 mL of an N-hexane:isopropyl alcohol:acetonitrile mixture (1:2:2, *v*/*v*) was added, vortexed for 30 s, and allowed to rest for 1 h. The mixture was then diluted with isopropyl alcohol:methanol (1:1, *v*/*v*) to a concentration of 0.2 mg/mL and prepared for HPLC–MS analysis.

##### HPLC–MS Analysis

The HPLC–MS system included a Thermo Fisher Scientific HPLC apparatus interfaced to a linear ion trap mass detector equipped with an atmospheric pressure chemical ionization (APCI) source (LTQ XL MS, Thermo Fisher Scientific, Waltham, MA, USA). Chromatographic separation was performed using a Thermo Scientific Hypersil GOLD C18 column (150 × 2.1 mm, 3 μm, Thermo Fisher Scientific, USA) using isopropyl alcohol and acetonitrile as the mobile phase. A gradient elution was applied during the analysis as follows: the isopropyl alcohol concentration was increased from 10 to 59% for 21 min, then rapidly increased from 59 to 90% in an instant, and finally held at 90% until 25 min had passed. The flow rate was 200 μL/min.

The APCI source was operated in the positive ionization mode. The dependent scan mode was applied. From the intensities of the top five fragment ions, we selected the parent ions for the next level collision. The optimized MS parameters were as follows: spray voltage of 4.0 kV, capillary temperature of 275 °C, source temperature of 300 °C, collision energy of 35 eV, and nitrogen as the sheath gas at a flow rate of 30 arbitrary units.

##### Identification and Quantification of TAGs

The acquired base peak chromatograms of TAGs and mass spectra obtained from LC-APCI-LIT/MS were exported as raw files by Xcalibur (Thermo Scientific, San Jose, CA, USA). TAGs identification and peak extraction were achieved using our previous study [46]. Mass signatures (*m*/*z* ratios) of parent and product fragment ions of TAGs were determined experimentally under data dependent second-level mass spectrometry (DDMS^2^) scan mode. The observed mass of the parent ion matched the mass of the triglyceride plus H^+^. Two or three major fragment ion species were observed and used for the identification of TAGs (Table 2).

Identified TAGs were quantified in fish oil samples using the mass spectrometry (MS) method with selected reaction monitoring (SRM). In the first stage of the mass scan, the mass spectrometer selects ions of one mass-to-charge ratio (the parent ions) and ejects all other ions from the mass analyzer. The scanning range of the secondary mass spectrum was set to contain the target product ion. So, two major fragment ions of the parent ion were received in the case of MS^2^. The larger fragment was used to specify the SRM transition, as it was detected at a higher intensity (Table 2). The SRM-MS scan included all parent–product pairs of TAGs reflecting the loss of one fatty acid. The quantification of TAGs was achieved using the peak area normalization method.

##### Identification and Quantification of EEs

Mass signatures (*m*/*z* ratios) of parent and product fragment ions were determined experimentally under direct injection using ethyl oleate (18:1 EE) as representative EEs. A parent ion (*m*/*z* 311.3) and two product fragment ions (*m*/*z* 283.3 and *m*/*z* 265.3) for 18:1 EE are acquired. The larger fragment ion (*m*/*z* 283.3) was 18:1 EE minus one CH_2_=CH_2_. The larger fragment ion (*m*/*z* 283.3) was used to specify the SRM transition, as it was detected at a higher intensity. Similar SRM transitions were calculated as described for 18:1 EE and used to detect additional EE species (Table 2). The SRM transition-quantified *m*/*z* 331.3→*m*/*z* 285.2 for Ethyl Eicosapentaneoate and *m*/*z* 357.3→*m*/*z* 311.2 for Ethyl Docosahexenoate with a dwell time of 200 ms for each transition were used during study sample analysis. Eight EEs were detected and quantified using the peak area normalization method.

## 3. Result and Discussion

### 3.1. Quantification of Fatty Acids in Fish Oil Using GC–MS

The fatty acids in the fish oils were detected based on the derivatization of fatty acids and their GC–MS analysis in the form of methyl esters. To quantify fatty acids in fish oil, we combined the SIM mode and equivalent chain length (ECL) values. The base peak ions of saturated, single unsaturated, double unsaturated, and polyunsaturated fatty acid methyl ester are *m*/*z* 74, 55, 67, and 79, respectively. Therefore, the SIM mode was set to *m*/*z* 55, 67, 74, and 79.

First, straight saturated FAMEs were identified by retention times of the FAMEs standard [32]. Second, the retention times of the straight saturated FAMEs found were used to calculate the ECL for unsaturated FAMEs according to Equation (1) in the Identification of Fatty Acids Section. Finally, the ECL of the FAMEs and characteristic ions for the fish oil were compared with those in available databases to identify the unsaturated FAMEs [47]. The quantification of fatty acids in fish oil was calculated according to the chromatographic peak area normalization method in SIM mode.

As shown in Table 3, 22 fatty acids in the fish oils were detected at levels >0.15% from the seven brands of fish oil samples. Fatty acids were predominantly unsaturated for all brands. However, the fraction percentages of saturated, monounsaturated, and polyunsaturated fatty acids showed only minor differences. Therefore, fatty acid (FA) compositions are not capable of distinguishing between TGFFO and EEFFO.

### 3.2. Detection of TAG_S_ in Fish Oil Using HPLC–MS

**Figure 1:**
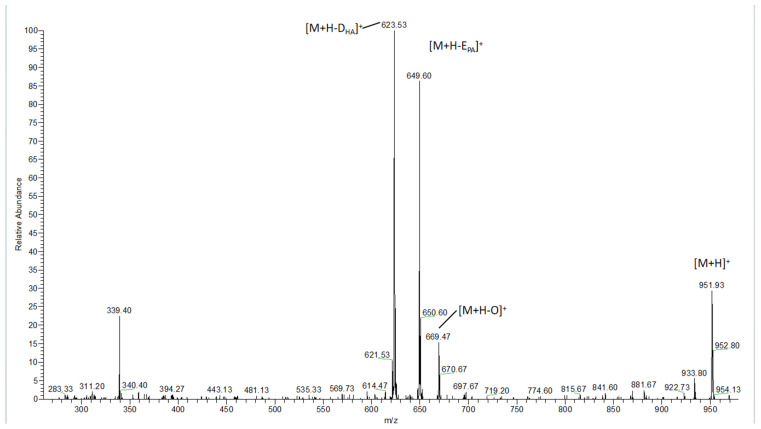
APCI-MS^2^ spectrum of [M+H]^+^ (*m/z* 951.7) for OE_PA_D_HA_ obtained from fish oil.

The TAGs in the fish oils were analyzed by reverse-phase liquid chromatography coupled with linear ion trap mass spectrometry (RPLC–LIT/MS), which has shown considerable potential using the APCI source. Quasi-molecular ions [M+H]^+^ were produced in positive mode for the APCI source. Figure 1 shows the collision-induced dissociation (CID) spectrum of quasi-molecular ion OE_PA_D_HA_ at *m*/*z* 951.7. In this MS^2^ spectrum, product ions at *m*/*z* 623.5, 649.6, and 669.4 were formed, indicating they are in the form of neutral losses from the fatty acids, including RCOOH of the docosahexaenoic (D_HA_) FA, the oleate (O) FA, and the eicosapntemacnioc (E_PA_) FA.

A quasi-molecular ion and three product ions of the TAGs of interest in the fish oils were compared with a customized database constructed in our previous study [46], and the quasi-molecular ion and three product ions of TAGs were determined as candidates. Using this method, 49 TAGs were identified in fish oils (Table 2). The relative abundance of each TAG ranged from a maximum value of approximately 41.86% to a minimum value of 1.09% (Table 4).

### 3.3. Detection of the Fatty Acid Ethyl Ester in Fish Oil Using HPLC–MS

**Figure 2:**
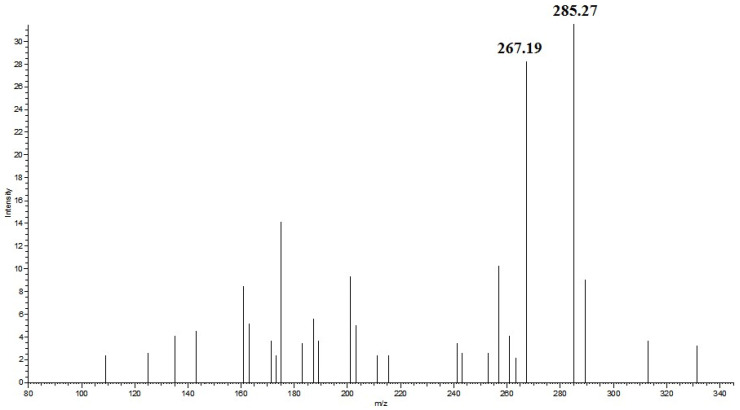
Positive-ion APCI mass spectra of ethyl eicosapentaenoate obtained from fish oil, The insert shows the MS^2^ spectrum of parent ion (*m/z*, 331.3) for ethyl eicosapentaenoate.

**Figure 3:**
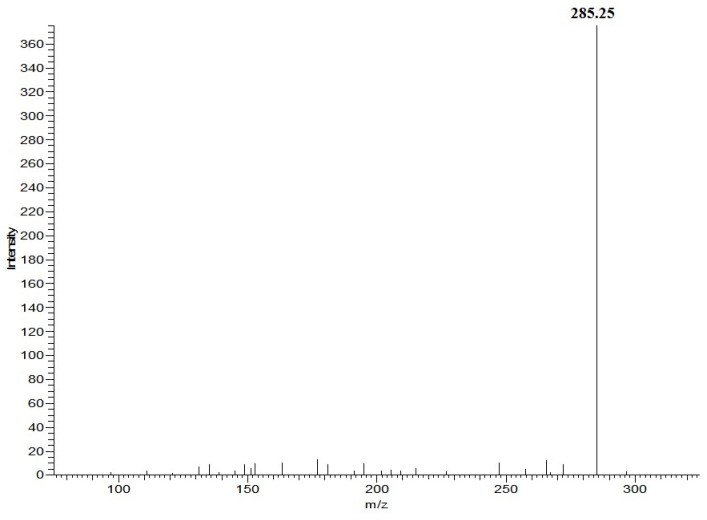
Positive-ion APCI mass spectra of ethyl stearate obtained from fish oil, The insert shows the MS^2^ spectrum of parent ion (*m/z*, 313.3) for ethyl stearate.

Through the analysis of the SRM chromatogram of fatty acids ethyl ester, it is found that the parent ion fragmentation mechanism of saturated and unsaturated fatty acids ethyl ester was different. It is not hard to find that the mass-to-charge ratio differs for the parent ion and the product ion in saturated and unsaturated fatty acids ethyl ester, which were 28 and 46, and according to the molecular structure, this may be due to neutral loss of CH_2_=CH_2_ and CH_3_CH_2_OH. The mass difference between the parent ion (*m*/*z* 331.3) and product ion (*m*/*z* 285.3) for ethyl eicosapentaenoate was due to the neutral loss of CH_3_CH_2_OH (Figure 2). The mass difference between the parent ion (*m*/*z* 313.3) and product ion (*m*/*z* 285.3) for ethyl stearate was due to the neutral loss of CH_2_=CH_2_ (Figure 3). Therefore, ethyl palmitate, ethyl stearate, ethyl oleate, ethyl arachidonate, ethyl eicosapentaenoate, ethyl docosapentaenoate, and ethyl docosahexaenoate were identified in EEFFO (Table 2).

### 3.4. Comparison of Components between TGFFO and EEFFO

A quantitative comparison of fatty acid components between TGFFO and EEFFO was conducted. The above analysis indicated that TGFFO and EEFFO were similar in terms of fatty acids and could be very difficult to distinguish (Table 3). Indeed, there is no significant difference among the content of major functional components DHA and EPA in different brands of fish oils. However, there is no doubt that the characteristic lipid compositions of TGFFO and EEFFO were greatly varied (Table 4). Seven chemical components, including ethyl eicosapentaenoate, ethyl docosapentaenoate, and other compounds, were screened as chemical markers for EEFFO. Triacylglycerols were not detected in EEFFO. Multiple triacylglycerol markers, including TG (16:1/18:2/20:5) and TG (18:0/18:1/22:6), have already been identified in TGFFO.

### 3.5. Fish Oil Determination Using Characteristic Lipid Fingerprints

**Figure 4:**
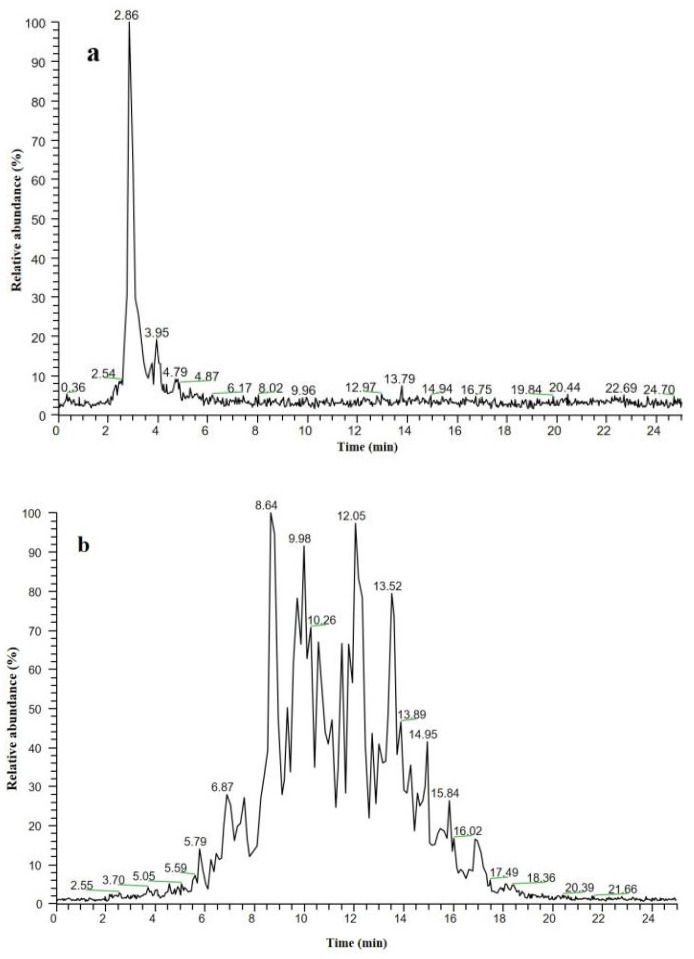
LC-APCI-IT/MS profiles of (**a**) ethyl ester fish oil and (**b**) triglycerides fish oil.

HPLC–MS is the most widely used technology in lipid analysis. There are significant differences between TGFFO and EEFFO samples in the total ion chromatogram of LC-APCI-LIT/MS, as shown in Figure 4. One chromatographic peak was observed for the EEFFO sample (Figure 4a). This peak at a retention time of 2.86 min, which overlaps the peaks of ethyl eicosapentaenoate and ethyl docosahexaenoate, was assigned as “characteristic peaks” of the EEFFO sample. The lipid separation results for TGFFO from LC-APCI-LIT/MS are shown in Figure 4b. TGFFO has many TAG chromatographic peaks like fingers at retention times of 6 min to 20 min. Therefore, it is relatively easy to differentiate between TGFFO and EEFFO using total ion chromatogram fingerprints.

## 4. Conclusions

Natural fish oil in triglyceride form is more expensive than artificial synthetic fish oil in ethyl ester form. Fish oil is particularly important for nutraceutical supplements; thus, determining the origins of fish oil is essential to detect any possible commercial fraud. This study showed that LC–LIT/MS analysis could be a valuable tool for this purpose. In this study, GC–MS and LC–APCI–LIT/MS methods for the determination of fatty acids and lipids were developed, respectively. Because GC–MS can only detect fatty acyl groups, it cannot distinguish between fish oil forms. In addition, the LC–APCI–LIT/MS method was successfully applied to distinguish between TGFFO and EEFFO. The LC–APCI–LIT/MS characteristics of EEFFO include neutral losses of CH_2_=CH_2_ (*m*/*z* 28) and CH_3_CH_2_OH (*m*/*z* 46). The LC–APCI–LIT/MS fingerprint chromatographic profile of TAG includes recognition features of the triglyceride form of fish oil. The assay was successfully applied to determine the forms of fish oil.

## Figures and Tables

**Table 1:** Description of the fish oils evaluated in this study.

Samples	Origin	Species
Fish oil 1	Peru	Sardines, Anchovies
Fish oil 2	South Pacific	Mackerel, Sardines, Anchovies
Fish oil 3	Norway	Tuna
Fish oil 4	Peru, Chile, Norway	Salmon, codfish
Fish oil 5	Arctic Ocean	codfish
Fish oil 6	Peru	Salmon
Fish oil 7	South Pacific	Salmon

**Table 2:** Identification of TAGs in deep sea fish oils.

	Lipid Name	Quantification Parent Ion MS^1^ [M+H]^+^ (*m/z*)	Quantification Product Ion MS^2^ (*m/z*)	Qualitative Product Ion MS^2^ (*m/z*)	Qualitative Product Ion MS^2^ (*m/z*)
1	TG(16:0 ^a^/14:0/16:1)	777.7	523.5	521.5	549.5
2	TG(16:0/12:0/18:1)	777.7	495.4	521.5	577.5
3	TG(16:0/14:0/16:2)	775.7	523.5	547.5	519.4
4	TG(16:1/14:0/16:2)	773.7	521.5	545.5	519.4
5	TG(16:1/14:0/17:0)	791.7	521.5	537.5	563.5
6	TG(15:0/16:2/16:2)	785.7	533.5	543.4	533.5
7	TG(16:0/16:0/16:1)	805.7	551.5	549.5	549.5
8	TG(16:0/16:1/16:1)	803.7	549.5	547.5	549.5
9	TG(16:1/16:1/16:1)	801.7	547.5	547.5	547.5
10	TG(16:1/17:0/17:0)	833.8	563.5	579.5	563.5
11	TG(18:1/14:0/18:1)	831.7	549.5	549.5	603.5
12	TG(16:0/14:0/20:5)	825.7	523.5	569.5	597.5
13	TG(16:1/14:0/20:5)	823.7	521.5	569.5	595.5
14	TG(16:2/14:0/20:5)	821.7	519.4	593.5	569.5
15	TG(15:0/16:0/20:5)	839.7	537.5	597.5	583.5
16	TG(12:0/20:5/20:5)	843.6	541.4	643.5	541.4
17	TG(16:0/18:1/18:1)	859.8	577.5	603.5	577.5
18	TG(18:0/14:0/20:4)	855.7	551.5	571.5	627.5
19	TG(16:1/14:0/22:6)	849.7	521.5	595.5	621.5
20	TG(12:0/20:5/22:6)	869.7	541.4	669.5	567.4
21	TG(18:0/16:1/20:4)	881.8	577.5	597.5	627.5
22	TG(16:0/18:1/20:5)	879.7	577.5	623.5	597.5
23	TG(16:1/18:1/20:5)	877.7	575.5	623.5	595.5
24	TG(16:1/18:2/20:5)	875.7	573.5	621.5	595.5
25	TG(16:1/16:1/22:6)	875.7	547.5	621.5	621.5
26	TG(17:0/17:0/22:5)	909.8	579.5	639.5	639.5
27	TG(16:0/18:1/22:5)	907.8	577.5	625.5	651.5
28	TG(18:1/18:1/20:5)	905.8	603.5	623.5	623.5
29	TG(16:1/18:2/22:6)	901.7	573.5	647.5	621.5
30	TG(15:0/20:5/22:6)	911.7	583.5	669.5	609.5
31	TG(17:0/18:1/22:6)	919.8	591.5	649.5	637.5
32	TG(18:1/20:5/20:5)	925.7	623.5	643.5	623.5
33	TG(14:0/22:6/22:6)	923.7	595.5	695.5	595.5
34	TG(16:2/20:5/22:6)	921.7	593.5	619.5	669.5
35	TG(18:0/18:1/22:6)	933.8	605.6	649.5	651.5
36	TG(18:0/20:5/22:6)	953.8	625.5	669.5	651.5
37	TG(18:1/20:5/22:6)	951.7	669.5	623.5	649.5
38	TG(16:0/22:6/22:6)	951.7	623.5	695.5	623.5
39	TG(20:5/20:4/20:5)	947.7	645.5	645.5	643.5
40	TG(17:0/22:6/22:6)	965.8	637.5	695.5	637.5
41	TG(18:1/22:6/22:6)	977.8	649.5	695.5	649.5
42	TG(20:5/20:5/22:5)	973.7	671.5	643.5	671.5
43	TG(18:3/22:6/22:6)	973.7	645.5	695.5	645.5
44	TG(20:5/20:5/22:6)	971.7	643.5	669.5	669.5
45	TG(20:5/22:6/22:6)	997.7	669.5	695.5	669.5
46	TG(22:5/22:6/22:6)	1025.8	697.5	695.5	697.5
47	TG(22:6/22:6/22:6)	1023.7	695.5	695.5	695.5
48	TG(17:0/20:5/22:6)	939.7	611.5	669.5	637.5
49	TG(18:0/22:5/22:6)	981.8	653.6	651.5	697.5
50	18:0 EE ^b^	313.3	285.3	267.3	- ^c^
51	18:1 EE	311.3	283.3	265.3	-
52	18:3 EE	307.3	279.2	261.2	-
53	18:4 EE	305.2	277.2	259.2	-
54	20:4 EE	333.3	305.2	287.2	-
55	20:5 EE	331.3	285.2	303.2	-
56	22:5 EE	359.3	331.3	313.3	-
57	22:6 EE	357.3	311.2	329.2	-

^a^ Numbers before and after a “:” mean number of carbon atoms and number of multiple double bonds present, respectively. ^b^ “EE” means ethyl ester. ^c^ “-” means there are not third identified fragment ion.

**Table 3:** FA levels in fish oil samples of seven brands. (Quantities of FAs are expressed as percentages (%), all samples were analyzed in triplicate).

FA Name	Fish Oil 1	Fish Oil 2	Fish Oil 3	Fish Oil 4	Fish Oil 5	Fish Oil 6	Fish Oil 7
	FA Levels (%) ± RSD (%)	FA Levels (%) ± RSD (%)	FA Levels (%) ± RSD (%)	FA Levels (%) ± RSD (%)	FA Levels (%) ± RSD (%)	FA Levels (%) ± RSD (%)	FA Levels (%) ± RSD (%)
12:0 ^a^	0.16 ± 4.8	n.d. ^f^	n.d.	n.d.	n.d.	n.d.	n.d.
14:0	11.93 ± 2	1.56 ± 5.5	11.55 ± 3.6	12.08 ± 3.2	11.88 ± 3.1	11.26 ± 2.5	4.66 ± 3.7
15:0	0.72 ± 4.7	0.29 ± 5	0.64 ± 6.3	0.69 ± 4.7	0.62 ± 5.1	0.59 ± 5.4	0.47 ± 5.5
16:0	26.62 ± 1.6	28.88 ± 1.7	26.91 ± 2.5	27.16 ± 5	26.21 ± 3.3	26.66 ± 1.8	26.43 ± 1.5
16:1n-9c ^b^	0.45 ± 4.8	0.36 ± 5.5	0.38 ± 6.7	0.38 ± 4.1	0.38 ± 6.8	8.46 ± 2.2	0.36 ± 5.6
16:1n-7c	9.18 ± 1.8	6.70 ± 3.1	8.14 ± 3	9.05 ± 3.7	8.33 ± 4	1.02 ± 7.8	5.62 ± 3.8
16:1n-5c	0.21 ± 5.3	n.d.	n.d.	n.d.	n.d.	n.d.	n.d.
16:2n-5c	1.10 ± 3	0.97 ± 6.8	1.09 ± 4.8	1.14 ± 4.6	1.16 ± 4.1	0.78 ± 6.9	0.81 ± 4.4
17:0	0.87 ± 4.3	1.43 ± 5.8	0.87 ± 4.9	0.80 ± 6	0.85 ± 7.1	1.09 ± 5.7	1.15 ± 5.9
16:3n-3c	1.67 ± 4.4	1.52 ± 5.9	1.68 ± 4.9	2.01 ± 3.1	2.21 ± 3.6	4.67 ± 4.4	1.40 ± 7.0
18:0	4.74 ± 3.3	6.42 ± 3.6	4.62 ± 4.3	4.59 ± 4.7	4.63 ± 6	8.53 ± 5.9	6.56 ± 7.6
18:1	8.38 ± 2.2	11.78 ± 3.6	8.73 ± 2.9	7.53 ± 5.7	7.67 ± 3.4	3.06 ± 4	10.13 ± 5.6
18:1n-9c	3.09 ± 3.7	4.26 ± 8.2	2.87 ± 4.6	2.99 ± 2.8	3.01 ± 5	1.22 ± 2	3.76 ± 2.6
18:1n-7c	0.40 ± 5.2	n.d.	n.d.	n.d.	n.d.	n.d.	n.d.
18:2 n-6	0.69 ± 4.8	1.82 ± 5.6	1.09 ± 4.1	1.04 ± 5.8	1.06 ± 5	0.64 ± 9	1.38 ± 3.0
18:3n-6c	2.33 ± 3.8	1.14 ± 6.1	0.66 ± 9	0.55 ± 8	0.54 ± 4.2	2.44 ± 3.2	0.44 ± 3.9
18:3n-3c	0.70 ± 5	3.87 ± 6	2.38 ± 5.3	2.35 ± 5.3	2.34 ± 3.3	0.72 ± 3.7	0.89 ± 4
20:1n-9c	0.22 ± 5.2	0.73 ± 5.6	0.45 ± 6.6	0.42 ± 5	0.40 ± 4.2	0.67 ± 7.7	1.83 ± 2.2
20:4n-3	0.70 ± 5.4	0.65 ± 6	0.59 ± 3.2	0.6 ± 4.1	0.63 ± 4.1	0.61 ± 6	0.75 ± 3.1
20:5n-3	16.07 ± 2.8	18.26 ± 3.8	16.87 ± 1.6	16.67 ± 5.5	18.57 ± 2.9	17.09 ± 1.9	22.15 ± 2.3
22:5n-3	1.42 ± 3.6	0.37 ± 5.2	0.46 ± 5.2	1.34 ± 6.8	0.53 ± 4.5	1.32 ± 3.6	0.56 ± 4.3
22:6n-3	7.04 ± 2.7	8.04 ± 3.3	8.72 ± 3.7	6.90 ± 4.9	7.58 ± 2.9	7.45 ± 3.5	9.78 ± 5.7
SFAs ^c^	45.03	38.57	44.59	45.32	44.19	48.14	39.28
MUFAs ^d^	21.92	23.83	20.57	20.38	19.80	14.43	21.69
PUFAs ^e^	31.73	36.64	33.53	32.61	34.62	35.71	38.16
EPA+DHA	23.12	26.30	25.59	23.58	26.15	24.53	31.92

^a^ Numbers before and after a “:” means number of carbon atoms and number of multiple double bonds present, respectively. ^b^ The number after “-” represents the position of double bond; c refers to cis-, while t to trans-. ^c^ “SFAs” means fatty acids. ^d^ “MUFs” means monounsaturated fatty acids. ^e^ “PUFAs” means polyunsaturated fatty acids. ^f^ “n.d.” means not detected.

**Table 4:** Lipid levels in fish oil samples of seven brands. (Quantities of Lipids are expressed as percentages (%), all samples were analyzed in triplicate. Those with relative abundance greater than 1% are listed in the table.).

	Lipid Name	Fish Oil 1	Fish Oil 2	Fish Oil 3	Fish Oil 4	Fish Oil 5	Fish Oil 6	Fish Oil 7
		Lipid Levels (%) ± RSD (%)	Lipid Levels (%) ± RSD (%)	Lipid Levels (%) ± RSD (%)	Lipid Levels (%) ± RSD (%)	Lipid Levels (%) ± RSD (%)	Lipid Levels (%) ± RSD (%)	Lipid Levels (%) ± RSD (%)
1	TG(16:0 ^a^/14:0/16:1)	10.19 ± 3.2	n.d. ^b^	n.d.	18.78 ± 5.6	n.d.	n.d.	n.d.
2	TG(16:0/12:0/18:1)	n.d.	n.d.	n.d.	n.d.	1.19 ± 5.8	n.d.	n.d.
3	TG(16:0/14:0/16:2)	n.d.	n.d.	n.d.	n.d.	7.24 ± 4.2	n.d.	n.d.
4	TG(16:1/14:0/16:2)	n.d.	n.d.	n.d.	n.d.	n.d.	2.35 ± 5.6	n.d.
5	TG(16:1/14:0/17:0)	n.d.	n.d.	n.d.	n.d.	1.09 ± 6.1	n.d.	n.d.
6	TG(15:0/16:2/16:2)	n.d.	n.d.	5.09 ± 5.3	n.d.	n.d.	n.d.	n.d.
7	TG(16:0/16:0/16:1)	15.95 ± 4.2	n.d.	n.d.	27.55 ± 5.4	n.d.	15.52 ± 3.1	n.d.
8	TG(16:0/16:1/16:1)	n.d.	n.d.	n.d.	n.d.	n.d.	15.84 ± 3.5	n.d.
9	TG(16:1/16:1/16:1)	n.d.	n.d.	n.d.	n.d.	n.d.	n.d.	7.19 ± 5.4
10	TG(16:1/17:0/17:0)	n.d.	n.d.	n.d.	n.d.	n.d.	15.19 ± 3.7	n.d.
11	TG(18:1/14:0/18:1)	n.d.	n.d.	n.d.	n.d.	n.d.	n.d.	21.55 ± 3.1
12	TG(16:0/14:0/20:5)	n.d.	n.d.	n.d.	n.d.	n.d.	n.d.	16.45 ± 3.5
13	TG(16:1/14:0/20:5)	n.d.	n.d.	n.d.	5.75 ± 3.1	n.d.	n.d.	n.d.
14	TG(16:2/14:0/20:5)	n.d.	n.d.	n.d.	n.d.	n.d.	7.45 ± 6.5	n.d.
15	TG(15:0/16:0/20:5)	n.d.	n.d.	n.d.	n.d.	n.d.	n.d.	1.26 ± 8.4
16	TG(12:0/20:5/20:5)	n.d.	n.d.	31.76 ± 3.6	n.d.	9.97 ± 3.9	n.d.	5.78 ± 7.6
17	TG(16:0/18:1/18:1)	n.d.	n.d.	n.d.	n.d.	n.d.	27.36 ± 3.5	n.d.
18	TG(18:0/14:0/20:4)	1.92 ± 5.9	n.d.	n.d.	n.d.	n.d.	n.d.	n.d.
19	TG(16:1/14:0/22:6)	n.d.	n.d.	n.d.	13.76 ± 3.2	n.d.	n.d.	n.d.
20	TG(12:0/20:5/22:6)	2.66 ± 3.7	n.d.	n.d.	n.d.	n.d.	n.d.	1.46 ± 8.7
21	TG(18:0/16:1/20:4)	n.d.	n.d.	n.d.	n.d.	n.d.	n.d.	2.12 ± 6.8
22	TG(16:0/18:1/20:5)	14.54 ± 4.1	n.d.	n.d.	n.d.	25.25 ± 3.4	n.d.	n.d.
23	TG(16:1/18:1/20:5)	n.d.	n.d.	n.d.	n.d.	34.14 ± 3.2	n.d.	n.d.
24	TG(16:1/18:2/20:5)	n.d.	n.d.	n.d.	6.11 ± 3.6	n.d.	5.70 ± 6.4	n.d.
25	TG(16:1/16:1/22:6)	11.42 ± 3.6	n.d.	n.d.	n.d.	n.d.	n.d.	n.d.
26	TG(17:0/17:0/22:5)	n.d.	n.d.	n.d.	n.d.	2.05 ± 6.5	n.d.	n.d.
27	TG(16:0/18:1/22:5)	6.16 ± 0.0	n.d.	n.d.	n.d.	n.d.	n.d.	n.d.
28	TG(18:1/18:1/20:5)	n.d.	n.d.	n.d.	n.d.	n.d.	n.d.	20.86 ± 2.3
29	TG(16:1/18:2/22:6)	10.01 ± 4.3	n.d.	n.d.	n.d.	n.d.	n.d.	n.d.
30	TG(15:0/20:5/22:6)	n.d.	n.d.	n.d.	1.27 ± 6.5	n.d.	n.d.	n.d.
31	TG(17:0/18:1/22:6)	1.73 ± 5.8	n.d.	n.d.	n.d.	n.d.	n.d.	n.d.
32	TG(18:1/20:5/20:5)	n.d.	n.d.	n.d.	n.d.	n.d.	3.50 ± 5.6	n.d.
33	TG(14:0/22:6/22:6)	4.13 ± 2.5	n.d.	n.d.	n.d.	n.d.	n.d.	n.d.
34	TG(16:2/20:5/22:6)	n.d.	n.d.	n.d.	n.d.	n.d.	4.32 ± 6.7	n.d.
35	TG(18:0/18:1/22:6)	n.d.	n.d.	41.86 ± 3.5	n.d.	n.d.	n.d.	n.d.
36	TG(18:0/20:5/22:6)	n.d.	n.d.	n.d.	n.d.	6.87 ± 3.5	n.d.	n.d.
37	TG(18:1/20:5/22:6)	n.d.	n.d.	n.d.	17.17 ± 5.4	n.d.	n.d.	11.04 ± 2.6
38	TG(16:0/22:6/22:6)	5.78 ± 3.8	n.d.	n.d.	n.d.	n.d.	n.d.	n.d.
39	TG(20:5/20:4/20:5)	n.d.	n.d.	n.d.	n.d.	2.92 ± 3.6	n.d.	n.d.
40	TG(17:0/22:6/22:6)	n.d.	n.d.	n.d.	n.d.	2.09 ± 6.3	n.d.	1.n.d.
41	TG(18:1/22:6/22:6)	3.36 ± 0.0	n.d.	n.d.	n.d.	n.d.	n.d.	2.22 ± 6.9
42	TG(20:5/20:5/22:5)	n.d.	n.d.	n.d.	n.d.	n.d.	n.d.	1.35 ± 7.2
43	TG(18:3/22:6/22:6)	n.d.	n.d.	n.d.	2.89 ± 3.5	n.d.	n.d.	n.d.
44	TG(20:5/20:5/22:6)	7.16 ± 3.4	n.d.	n.d.	n.d.	n.d.	n.d.	6.50 ± 3.5
45	TG(20:5/22:6/22:6)	2.35 ± 2.5	n.d.	19.13 ± 3.1	4.61 ± 1.5	5.39 ± 3.1	n.d.	n.d.
46	TG(22:5/22:6/22:6)	n.d.	n.d.	2.13 ± 5.8	n.d.	n.d.	n.d.	n.d.
47	TG(22:6/22:6/22:6)	n.d.	n.d.	n.d.	n.d.	n.d.	1.32 ± 6.9	n.d.
48	TG(17:0/20:5/22:6)	1.00 ± 6.8	n.d.	n.d.	n.d.	n.d.	n.d.	n.d.
49	TG(18:0/22:5/22:6)	n.d.	n.d.	n.d.	1.00 ± 5.6	n.d.	1.00 ± 8.1	n.d.
50	18:0 EE ^c^	n.d.	5.04 ± 4.6	n.d.	n.d.	n.d.	n.d.	n.d.
51	18:1 EE	n.d.	3.54 ± 6.7	n.d.	n.d.	n.d.	n.d.	n.d.
52	18:3 EE	n.d.	1.38 ± 6.4	n.d.	n.d.	n.d.	n.d.	n.d.
53	18:4 EE	n.d.	9.09 ± 3.5	n.d.	n.d.	n.d.	n.d.	n.d.
54	20:4 EE	n.d.	3.06 ± 5.9	n.d.	n.d.	n.d.	n.d.	n.d.
55	20:5 EE	n.d.	42.87 ± 2.5	n.d.	n.d.	n.d.	n.d.	n.d.
56	22:5 EE	n.d.	4.10 ± 5.1	n.d.	n.d.	n.d.	n.d.	n.d.
57	22:6 EE	n.d.	30.94 ± 3.2	n.d.	n.d.	n.d.	n.d.	n.d.

^a^ Numbers before and after a “:” mean number of carbon atoms and number of multiple double bonds present, respectively. ^b^ “n.d.” means not detected. ^c^ “EE” means ethyl ester.

## Data Availability

The original contributions presented in the study are included in the article, further inquiries can be directed to the corresponding author.
